# Platelet-to-lymphocyte ratio is correlated with a delay in feeding resumption following a transhiatal esophagectomy with cervical anastomosis

**DOI:** 10.1186/s12957-020-02035-y

**Published:** 2020-10-14

**Authors:** Antoine El Asmar, Elie Ghabi, Toufic Saber, Christina Abou-Malhab, Bernard Akl, Ziad El Rassi

**Affiliations:** 1 Institut Jules Bordet, Boulevard de Waterloo 121, 1000 Brussels, Belgium; 2 Department of Urology, Saint George Hospital University Medical Center, P.O. Box 166378, Achrafieh, Beirut, 1100 2807 Lebanon; 3 Department of General Surgery, Saint George Hospital University Medical Center, P.O. Box 166378, Achrafieh, Beirut, 1100 2807 Lebanon; 4 Faculty of Medicine and Medical Sciences, University of Balamand, P.O. Box 166378, Achrafieh, Beirut, 1100 2807 Lebanon

**Keywords:** Neutrophil-to-lymphocyte ratio, Platelet-to-lymphocyte ratio, Prognostic tool

## Abstract

**Introduction:**

The lymphocytic population, neutrophil-to-lymphocyte ratio (NLR), and platelet-to-lymphocyte ratio (PLR) are prognostic tools predictive of adverse outcomes for several solid tumors and oncologic surgeries, one of which is esophageal adenocarcinoma. Furthermore, delayed resumption of oral feeding postoperatively is associated with significant morbidity. Given the controversies regarding post-op nutritional support in these patients, this study investigates the prognostic role of the lymphocytic percentage, the NLR, and the PLR in predicting prolonged length of hospital stay (LOHS) and ICU stay (LOICUS) as well as delayed oral feeding following transhiatal esophagectomy (THE) for adenocarcinoma of the esophagogastric junction (AEG).

**Methods:**

Forty consecutive patients who underwent transhiatal esophagectomy performed by a single surgeon for Siewert type II and type III adenocarcinoma of the esophagogastric junction at a tertiary referral center were selected. Retrospective data collection was performed from the patients’ medical records, and statistical analysis was performed using Pearson correlation and Student’s *t* test and Chi-square testing.

**Results:**

An increased LOHS was correlated with a lower preoperative lymphocyte percentage (*p = 0.043*), higher NLR (*p = 0.010*) and PLR (*p = 0.015*), and an increased number of packed red blood cell (PRBC) transfusions perioperatively (*p = 0.030*). An increased LOICUS was correlated with a lower preoperative lymphocyte percentage (*p = 0.033*), higher NLR (*p = 0.018*) and PLR (*p = 0.044*), an increased number of PRBC transfusions (*p = 0.001*), and patients’ comorbidities (*p < 0.05*). A delay in feeding resumption was correlated with a lower preoperative lymphocyte percentage (*p = 0.022*), higher NLR (*p = 0.004*) and PLR (*p = 0.001*), an increased PRBC transfusions (*p = 0.001*), and diabetes mellitus (*p = 0.033*). Multivariate analysis with automatic linear modeling showed that only the preoperative PLR was a powerful predictor for the delay of feeding resumption (*p* < 0.01).

**Conclusion:**

The lymphocyte percentage, PLR, and NLR are found to be associated with prolonged hospitalization and ICU stay and delayed oral feeding following THE for Siewert types II and III AEG. We hope by this series, to have set, at least one preliminary cornerstone, in the creation of a prognostic model, capable of assessing the need for an intraoperative jejunostomy placement, in patients undergoing esophagectomy for distal esophageal carcinoma.

## Background

With a steadily increasing incidence and a stable dismal prognosis [1, 2], adenocarcinoma of the esophagogastric junction (AEG) remains to be a significant clinical entity requiring prompt surgical intervention and orchestrated care from a multidisciplinary team. Transhiatal esophagectomy (THE) is one such intervention which is best performed in tertiary referral centers due to its technical difficulty and high postoperative morbidity.

Tumor-associated inflammation is an emerging field of interest and debate due to its implications on tumor progression and, subsequently, patient prognosis [3]. The association between such factors and multiple solid tumors has been investigated, with numerous studies done on AEG [4–7]. The conclusions of these studies, however, remain debatable. Lymphocyte percentage, the neutrophil-to-lymphocyte ratio (NLR), and the platelet-to-lymphocyte ratio (PLR), which can be obtained from routine hematologic bloodwork, are markers of clinical and subclinical inflammation that have been both shown to be predictive of adverse perioperative outcomes in some studies and of no statistically significant utility in other studies [4, 6, 7].

Delayed oral feeding following an esophagectomy has been shown to directly impact the length of hospital stay (LOHS), return of bowel movement, and return to baseline functioning as well as overall oncologic outcomes [8]. Therefore, many advocates for early oral feeding, often as early as 48 h post-operatively, have demonstrated the efficacy and safety of this practice when compared to established protocols such as early enteral feeding and total parenteral nutrition [9]. These feeding regimens, however, are not universal. Furthermore, given both the controversy regarding the mode of nutritional support following an esophagectomy, particularly with the common practice of routine placement of feeding jejunostomies [10], as well as the demonstrated prognostic role of inflammatory biomarkers, the role of the NLR and PLR in predicting delayed oral feeding must be demonstrated to best identify patients who may benefit from having a feeding jejunostomy placed for optimal nutritional support.

In order to assess the clinical significance and prognostic utility of these biomarkers to contribute to the growing effort in improving patient quality of care and anticipating adverse outcomes in patients undergoing major abdominal oncologic surgery, we performed a retrospective analysis of a consecutive series of patients who underwent THE for Siewert type II and type III AEG by a single surgeon at our institution over an 8-year period. The choice of a single surgeon sample serves to control for surgical technique and operator bias. Furthermore, to further validate our results, we have included known predictors of postoperative morbidity as analytical controls in our analysis.

## Methods

### Sample selection

A sample of 40 consecutive patients who underwent THE and cervical esophagogastric anastomosis with curative intent for AEG by a single surgeon at Saint Georges Hospital University Medical Center (SGHUMC), a tertiary referral center, between 2012 and 2018 was identified. The decision to limit the study to single surgeon’s experience is due to three factors. First, the operative load of the selected surgeon is the greatest at our referral center whereas the contributions of the other surgeons in practice were not significant. These cases were excluded to reduce the variability in surgical technique and minimize its influence on postoperative outcomes. Therefore, the choice of a single surgeon with a high operative load serves to minimize the influence of potential operator-dependent confounders and, subsequently, improve the power and reliability of the results and analysis.

The following inclusion criteria were described:

- Pathology: adenocarcinoma of the distal esophagus or gastric cardia
- Classification: Siewert types II and III
- Type of intervention: transhiatal esophagectomy with cervical anastomosis, no feeding jejunostomy was placed during the procedure
- Intent: curative
- Neoadjuvant treatment: chemoradiotherapy for type II, chemotherapy for type III, and no neoadjuvant therapy for T1b or T2, N0 tumors
- Preoperative diagnosis and staging: gastroscopy with biopsy, computed tomography (CT), and positron emission tomography (PET) scans with follow-up staging preoperatively after neoadjuvant treatment
- Preoperative preparations: no chemotherapy or radiotherapy and no blood product transfusions, at least 1 month before surgery to avoid any influence neoadjuvant regimens may have on a preoperative complete blood count and so the PLR and NLR [11]

### Data collection

Data collection was performed retrospectively from the patients’ medical records. Demographic, operative and perioperative, and biochemical data were collected. Pertinent demographic information included the patient’s comorbidities, namely the presence of comorbid diabetes mellitus (DM), hypertension (HTN), and cardiovascular disease (CVD). Perioperative information included the length of ICU stay (LOICUS), length of hospital stay (LOHS), day of feeding resumption, number of postoperative PRBC transfusions, and the occurrence of clinically significant postoperative complications requiring medical or surgical intervention. Pertinent laboratory biomarkers included the preoperative complete blood count (CBC). The PLR was calculated by dividing the absolute platelet count by the absolute lymphocyte count. The NLR was calculated in a similar fashion. Data input was performed using Microsoft Excel version 1908.

### Statistical analysis

Given the small sample size of this study, tests of normality were not performed. Pearson correlation was performed to determine if a correlation exists between two continuous variables. Student’s *t* test was used for continuous variables, and Chi-square was used for categorical variables. Multivariate analysis was performed using multiple linear regression. Statistical significance was defined by a *p* value less than 0.05. The statistical analysis was performed using SPSS version 24.0.

## Results

The sample was composed of 40 patients, 26 of whom were males (65%) and 14 of whom were females (35%). Nine of these patients received neoadjuvant chemotherapy alone, and 3 patients received neoadjuvant chemotherapy and radiotherapy 1 month prior to surgery. None of these patients received any neoadjuvant treatment within 1 month of surgery in order to eliminate the influence of treatment on the preoperative CBC and so the lymphocyte percentage, PLR, and NLR, as well as other preoperative labs [11]. The average number of comorbidities was 2.2 with comorbid diabetes present in 25% of patients, hypertension in 37.5%, and cardiovascular disease in 42.5%. 12.5% had a second primary malignancy at the time of presentation. Fifty-five percent of patients had a history of smoking.

The median operative time was 255 min (interquartile range (IQR) = 1.1) with a minimum operative time of 180 min and a maximum of 420 min. The median day of oral feeding resumption was 8 days with a minimum of 7 days and a maximum of 19 days. The median LOICUS was 3 days (IQR = 4.5) with a maximum stay of 22 days observed for 1 patient and a minimum stay of 0 days observed for 4 patients. The median LOHS was 14 days (IQR = 6.25) with a maximum stay of 45 days for 1 patient and a minimum of 9 days for 1 patient.

Eighteen patients (47.4%) developed postoperative complications. Mild complications (Clavien-Dindo grade 1) were observed in 12 patients (30%). Major complications (Clavien-Dindo grade 2, 3, or 4) were seen in 6 patients (15%). Of these patients, 2 developed a cervical anastomotic leak that required no surgical intervention. It is worth noting that 1 patient developed pneumonia and ARDS post-operatively and, due to a prolonged ICU stay of 15 days, required a feeding jejunostomy for nutritional support.

### Lymphocyte percentage

The mean preoperative lymphocyte percentage was 24.98%. Pearson correlation revealed a negative correlation between lymphocyte percentage (Pearson coefficient = − 0.365, *p* value = 0.043) and LOHS. Similarly, a negative association existed for LOICUS (Pearson coefficient = − 0.385, *p* value = 0.033) and day of oral feeding resumption (Pearson coefficient = 0.423, *p* value = 0.022).

### Neutrophil-to-lymphocyte ratio

**Fig. 1:**
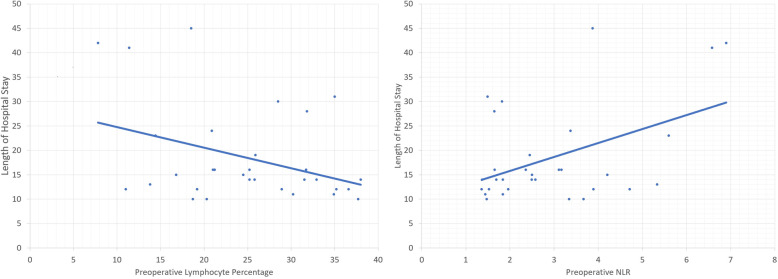
Correlation between preoperative lymphocyte percentage, neutrophil-to-lymphocyte ratio and length of hospital stay

**Fig. 2:**
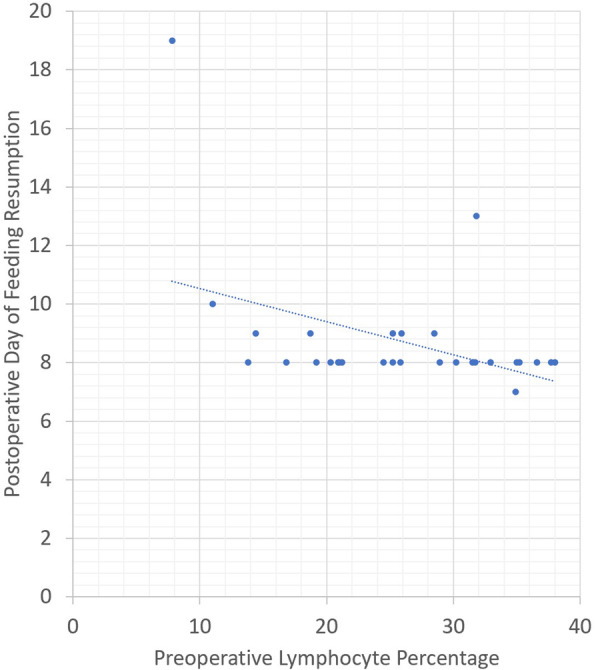
Correlation between preoperative lymphocyte percentage and postoperative day of feeding resumption

**Fig. 3:**
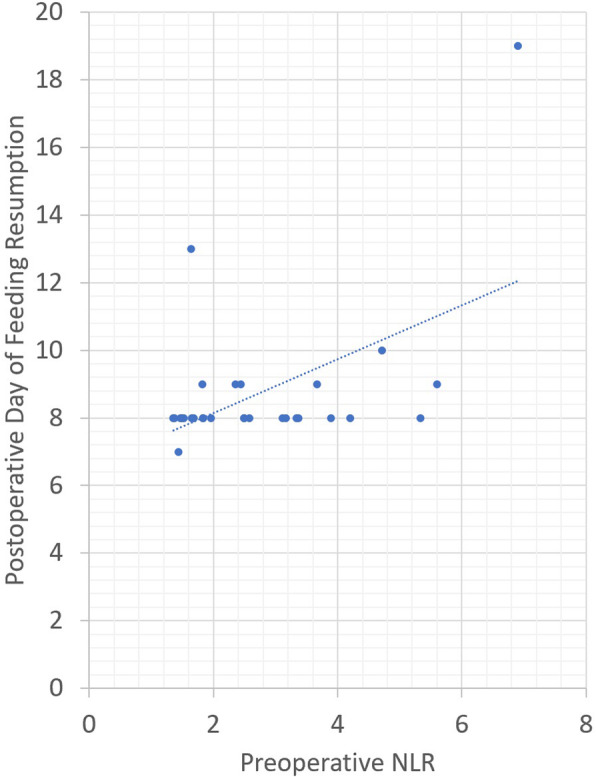
Correlation between preoperative NLR and postoperative day of feeding resumption

The average preoperative NLR was 2.94. Pearson correlation revealed that a higher preoperative NPR was positively correlated with an increase in LOHS (Pearson coefficient = 0.454, *p* value = 0.01) and LOICUS (Pearson coefficient = 0.422, *p* value = 0.018) and delayed resumption of oral feeding (Pearson coefficient = 0.517, *p* value = 0.004). Figure 1 demonstrates the association between LOICUS and preoperative lymphocyte percentage as well as LOICUS and preoperative NLR. Figure 2 demonstrates the association between postoperative day of feeding resumption and the preoperative lymphocyte percentage. Figure 3 demonstrates the association between postoperative day of feeding resumption and the NLR.

### Platelet-to-lymphocyte ratio

**Fig. 4:**
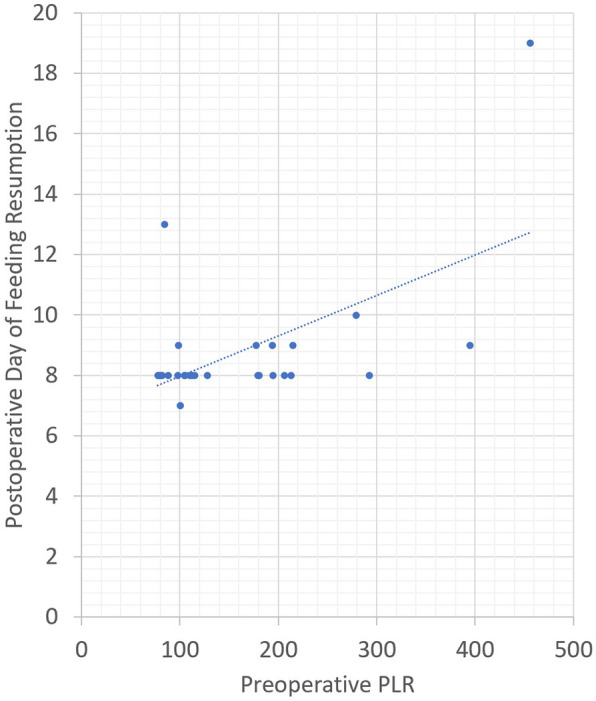
Correlation between PLR and postoperative day of feeding resumption

The average preoperative PLR was 165.6. Similar to the pre-op NLR, Pearson correlation revealed that a higher preoperative PLR was positively correlated with an increase in LOICUS (Pearson coefficient = 0.364, *p* value = 0.044) and delayed resumption of oral feeding (Pearson coefficient = 0.578, *p* value = 0.001). A correlation between pre-op PLR and LOHS was not statistically significant (Pearson coefficient = 0.284, *p* value = 0.12). Figure 4 demonstrates the association between postoperative day of feeding resumption and the PLR.

### Perioperative packed red blood cell transfusions

Patients received a median of 1 transfusion (IQR = 2.5) and an average of 1.625 transfusions of PRBCs. The number of PRBC transfusions was found to be positively correlated with an increase in LOHS (Pearson coefficient = 0.383, *p* value = 0.03) and LOICUS (Pearson coefficient = 0.597, *p* value < 0.001) and a delayed resumption of oral feeding (Pearson coefficient = 0.566, *p* value < 0.001).

### Patient comorbidities

The presence of DM, HTN, and CVD was correlated with and increased LOICUS (*p value = 0.022*, *0.011*, and *0.028* respectively), but only DM was correlated with a delay in feeding resumption (*p value = 0.033*). Table 1 demonstrates the correlation of patients’ comorbidities with LOICUS in days.

**Table 1:** Correlation of patients’ co-morbidities with the LOICUS in days

		Number of patients	Mean duration of ICU stay in days	Std. deviation	Std. error mean	*p* value
Diabetes	Absence	23	3.30	2.619	.546	*0.022*
	Presence	9	7.22	6.723	2.241	
Hypertension	Absence	17	2.59	2.293	.556	*0.011*
	Presence	15	6.47	5.397	1.393	
Cardiovascular disease	Absence	15	2.60	2.384	.616	*0.028*
	Presence	17	6.00	5.244	1.272	

### Univariate analysis

Univariate analysis showed that lymphocyte percentage, NLR, PLR, PRBCs, and patients’ comorbidities were all correlated with an increased LOHS and LOICUS and a delay in feeding resumption. All were statistically significant with *p < 0.05*. The correlation between operative time and LOHS, LOICUS, and delay in oral feeding resumption was, however, not statistically significant.

### Multivariate analysis

On multivariate analysis, a delay of feeding resumption is shown to be the most powerful predictor of increased LOHS (*p* value < 0.01) and LOICUS (*p* value < 0.01). In turn, LOICUS (*p* value < 0.01) and pre-op PLR (*p* value < 0.01) are found to be the most powerful predictors of a delay in resumption of oral feeding. Moreover, multivariate analysis did not demonstrate a statistically significant correlation between operative time, LOHS, LOICUS, and delay in feeding resumption.

## Discussion

The complex interaction between neoplastic cells and the host’s inflammatory response, represented by biochemical and hematological tests and ratios, has been investigated in various studies [12–15] though the molecular mechanism behind the demonstrated associations is not yet entirely understood. Some authors argue that the paraneoplastic inflammatory response contributes to patient’s catabolic state [16, 17] and, ultimately, cancer cachexia. With that in mind, the role of biochemical markers such as C-reactive protein (CRP) and albumin [18, 19], which are established markers of inflammation and nutritional status, in the systemic inflammatory response has been investigated to devise certain prognostic scores to predict a patient’s clinical outcome [20]. The Glasgow Prognostic Score, which uses CRP and albumin, is one such example validated for colorectal and esophageal cancer [21–26].

The prognostic utility of the NLR and PLR in colorectal cancer has been demonstrated by Guthrie et al. whereby elevated ratios are indicative of a higher likelihood of postoperative complications [27]. Overall morbidity in head and neck surgery is also reflected by elevated PLR and NLR, as reported by Maruyama et al. [28]. In esophageal cancer, Vulliamy et al. demonstrated that NLR can predict post-esophagectomy complications [29]; however, Testumi et al. refuted such a claim [7].

With this in mind, the role of the lymphocyte percentage, NLR, and PLR, which are inexpensive and readily available biomarkers, in predicting unfavorable outcomes and decreased overall survival becomes more apparent, particularly when high values have been shown to be associated with deep tumor invasion and nodal metastasis of esophageal adenocarcinoma [7] as well as increased likelihood of complications following an esophagectomy.

The mechanism behind the NLR and PLR ratios has not yet been fully elucidated. Lymphocytes have been demonstrated to have a positive role in managing tumor cells whereby increased tumor infiltration mediated by lymphocytes is associated with improved response to chemotherapy and overall prognosis [30]. The leukocyte and cytokine environment has also been demonstrated to impact tumor progression and lymphocyte antitumor activity. Neutrophils are capable of inhibiting an antitumor response by suppressing lymphocyte activity as well as activated T cell and NK cell activity [30]. Furthermore, a high peritumoral macrophage environment, as reflected by the NLR, is associated with higher levels of interleukin 17 as well as other cytokines. This, along with neutrophil and macrophage-derived secretion tumor growth factors such as hepatocyte growth factor, IL-6, IL-8, and metalloproteases, the tumor microenvironment becomes stimulated [30]. Furthermore, a decrease in the number of lymphocytes reflects a decrease in the host’s immune system ability to elicit antibody-dependent cell-mediated cytotoxicity, thus slowing down tumor’s progression and metastasis [4, 31]. This proposed mechanism is observed in our study as demonstrated by the protective association between lymphocyte percentage, and therefore absolute lymphocyte count, with hospitalization, ICU stay, and commencement of oral feeding as compared to the adverse association seen between the NLR and PLR and the same outcomes.

This study is not without limitations. The biggest limiting factor is the small sample size. This, however, is mainly due to a rigorous selection process which aims to reduce operator and technical bias. To minimize operator-dependent and technique-related biases associated to postoperative morbidity, we only included patients with Siewert II and III tumors, operated by the same surgeon, using the same technique and with a curative intent.

Furthermore, given that patient comorbidities have been established to reflect a poor postoperative course [32] by using variables with established associations to serve as an analytical control when testing the ratio association with adverse outcomes, we aim to address the size limitation of this study. Preoperative patients’ comorbidities are known factors in predicting post-esophagectomy complications. These complications lead to a marked increase in the LOHS [32]. In our study, we integrated these comorbidities in the analysis process, trying to identify if lymphocytic volume, NLR, and PLR can also predict the emergence of postoperative adverse events. As shown by our univariate analysis, lymphocyte percentage, NLR, PLR as well as perioperative PRBCs transfusion and patients’ comorbidities predict a longer ICU stay, hospital stay, and a delay in feeding resumption. These outcomes reflect a higher postoperative complication burden. Further multivariate analysis demonstrated the significance of PLR in delaying feeding resumption.

Moreover, more research is required to test these biomarkers and ratios to further expand upon their prognostic utility and to better elucidate the mechanisms responsible for these associations, particularly in patients with esophageal adenocarcinoma. Lastly, given that these biomarkers mirror the variables used in devising the risk adjustment model devised by the Society of Thoracic Surgeons General Thoracic Surgery Database [32], the importance of these variables becomes apparent in developing a risk assessment model for post-esophagectomy morbidity.

## Conclusion

An increased platelet-to-lymphocyte ratio is correlated with a delay in feeding resumption following a transhiatal esophagectomy with cervical anastomosis. Such a finding will allow us to further investigate, on a larger scale, the value of these biological markers in predicting prognosis, and to incorporate additional outcomes defining postoperative morbidity, such as the length of hospital and ICU stay, and the delay in feeding resumption. Devising such a score might enable surgeons in the future to classify patients’ risk and consequently predict the need for an intraoperative feeding jejunostomy, closer postoperative monitoring, or devising more rigorous preventive surveillance measures.

## Data Availability

The datasets generated and/or analyzed during the current study are not publicly available due to them being collected within the institution but are available from the corresponding author on reasonable request.

## Abbreviations

NLR: Neutrophil-to-lymphocyte ratio
PLR: Platelet-to-lymphocyte ratio
LOHS: Length of hospital stay
LOICUS: Length of ICU stay
THE: Transhiatal esophagectomy
AEG: Esophagogastric junction
PRBCs: Packed red blood cells
SGHUMC: Saint George Hospital University Medical Center
CT: Computed tomography
PET: Positron emission tomography
DM: Diabetes mellitus
HTN: Hypertension
CVD: Cardiovascular disease
CBC: Complete blood count
IQR: Interquartile range
